# Multi-Layer Nanofibrous PCL Scaffold-Based Colon Cancer Cell Cultures to Mimic Hypoxic Tumor Microenvironment for Bioassay

**DOI:** 10.3390/cancers13143550

**Published:** 2021-07-15

**Authors:** Eun-Taex Oh, Ha Gyeong Kim, Min-Ho Choi, Jae-Seon Lee, Sang Jeong Kim, Jong-Young Kwak, Heon Joo Park

**Affiliations:** 1 Department of Biomedical Sciences, College of Medicine, Inha University, Incheon 22212, Korea; nbstoet@inha.ac.kr; 2 Program in Biomedical Science & Engineering, Inha University, Incheon 22212, Korea; hagyeong315@inha.edu (H.G.K.); jaeslee@inha.ac.kr (J.-S.L.); 3 Department of Biomedical Sciences, The Graduate School, Ajou University, Suwon 16499, Korea; minho8068@ajou.ac.kr; 4 Immune Network Pioneer Research Center & 3D Immune System Imaging Core Center, Ajou University, Suwon 16499, Korea; 5 Department of Molecular Medicine, College of Medicine, Inha University, Incheon 22212, Korea; 6 Research Center for Controlling Intracellular Communication, College of Medicine, Inha University, Incheon 22212, Korea; 7 Department of Physiology, Seoul National University College of Medicine, Seoul 03080, Korea; sangjkim@snu.ac.kr; 8 Department of Microbiology, College of Medicine, Inha University, Incheon 22212, Korea

**Keywords:** cancer cell culture, hypoxia, nanofibrous scaffold, PCL, three-dimensional

## Abstract

**Simple Summary:**

Multi-layer, nanofibrous poly(ε-caprolactone) (PCL) scaffold (pNFS)-based colon cancer cell cultures mimic the hypoxic tumor microenvironment. The simple procedure generates a 3D hypoxic tumor microenvironment comprising defined numbers and densities of colon cancer cells with easily controllable lateral dimensions and a thickness defined by pNFS. This pNFS-based multi-layered colon cancer cell culture system is useful for bioassays, for drug screening, and as a replacement for small animals in testing the effects of a hypoxic tumor microenvironment.

**Abstract:**

Three-dimensional (3D) cancer cell culture systems have been developed to aid the study of molecular mechanisms in cancer development, identify therapeutic targets, and test drug candidates. In this study, we developed a strategy for mimicking the hypoxic tumor microenvironment in a 3D cancer cell culture system using multi-layer, nanofibrous poly(ε-caprolactone) (PCL) scaffold (pNFS)-based cancer cell cultures. We found that human colon cancer cells infiltrated pNFS within 3 days and could be cultured three-dimensionally within the NFS. When incubated in four stacks of 30 µm-thick pNFS for 3 days, colon cancer cells in layer three showed partially reduced entry into the S phase, whereas those in layer four, located farthest from the media, showed a marked reduction in S-phase entry. As a consequence, cells in layer four exhibited hypoxia-induced disorganization of F-actin on day 3, and those in layers three and four showed an increase in the expression of the hypoxia-induced transcription factor HIF-1α and its target genes, *Glut1*, *CA9*, *VEGF*, and *LDHA*. Consistent with these results, doxorubicin- and ionizing radiation-induced cell death was reduced in colon cancer cells cultured in layers three and four. These results suggest that pNFS-based multi-layer colon cancer cell cultures mimic the hypoxic tumor microenvironment and are useful for bioassays.

## 1. Introduction

Three-dimensional (3D) cell culture systems represent the real microenvironment in which cells exist in tissues more accurately than two-dimensional (2D) cell culture systems [1,2,3,4,5,6]. Thus, 3D cell culture systems are of increasing interest to investigate human biology and drug discovery due to their advantages of providing more physiological features [1,2,3,4,5,6,7].

Cell spheroid cultures have been used as 3D culture systems for investigating metastasis and invasion, performing pharmacological assays of cancer cells and, in general, for mimicking in vivo conditions [4,8,9,10]. However, it remains difficult to accurately reconstruct the precise 3D locations of many types of cells in such spheroid cultures owing to cellular heterogeneity, the inability to control cell numbers, and necrosis caused by a lack of nutrients [4]. Various types of 3D scaffolds consisting of fabrics of packed fibers with a high surface density that promotes cell attachment, proliferation, and movement on the surface have been developed [11,12]. Alternative multi-layered 3D cell culture systems, such as polymer-based mesh, mesh-like hydrogel sheets, paper-supported gels, polystyrene, and hydrogel, have also been developed to improve 3D culture systems and mimic tumor microenvironments [1,13,14,15,16,17,18]. However, 3D culture systems have certain disadvantages. For example, cells in each layer are separated from each other, making it difficult to detect intercellular interactions between layers; cell density is not as high as the actual density in living tissue; and specific instrumentation that is not commonly available in biological laboratories is often required.

Microfabrication techniques capable of creating architecturally complex scaffolds for 3D cell culture systems have been developed [1,11,19]; however, most of these techniques require specialized instrumentation that is not typically found in a research setting. Recently, mats consisting of fabricated and patterned biodegradable nanofibrous scaffolds (NFS), which are readily available in biological laboratories, have been developed for cell culture [11,20,21]. As cells generally grow poorly inside an NFS, it is necessary to develop a type of NFS that mimics the structure of natural ECM and thus is suitable for 3D culture of cells [11]. In a previous report, we developed nanofibrous poly(ε-caprolactone) (PCL) mats (pNFS) consisting of nanoscale fibers (400–800 nm in diameter) and submicron-scale fibers (1–2 µm in diameter) [11]. This pNFS provides sufficient cell infiltration to support a stable 3D structure and was shown to promote 3D adhesion, spreading, migration, and activity of dendritic cells in coculture with cancer cells [11].

In solid tumors, cancer cells are exposed to a continuum of oxygen concentrations that reflects their distance from capillaries [22]. As the distance from capillaries increases, oxygen is depleted, and the tumor cells become necrotic [22]. In this hypoxic area, where tumor cells probably form layers one or two cells thick, the oxygen concentration is high enough for the cells to survive but low enough to provide relative protection against the effects of chemotherapy or radiotherapy [22].

In this study, we present a strategy for mimicking the 3D hypoxic tumor microenvironment using a multi-layer pNFS-based colon cancer cell culture system. This simple procedure generates a 3D hypoxic tumor microenvironment comprising defined numbers and densities of cancer cells with easily controllable lateral dimensions and a thickness defined by NFS. In addition, such a multi-layer pNFS-based colon cancer cell culture system allows for easy observation of the biological properties of cells at the desired tissue depth. Notably, colon cancer cells in the hypoxic layer showed increased expression of the hypoxic marker, HIF-1α (hypoxia-induced factor 1α), and enhanced chemo- and radioresistance. Collectively, our findings demonstrate that this pNFS-based multi-layered colon cancer cell culture system is useful for bioassays, drug screening, and as a replacement for small animals in testing the effects of a hypoxic tumor microenvironment.

## 2. Materials and Methods

### 2.1. Cell Lines and Culture Conditions

HCT116 human colon cancer cells and HeLa human cervical cancer cells were obtained from American Type Culture Collection (Manassas, VA, USA) and cultured in Dulbecco’s Modified Eagle Medium (Invitrogen, Carlsbad, CA, USA) supplemented with 10% fetal bovine serum (FBS; Invitrogen, Carlsbad, CA, USA) and 1% antibiotics (Invitrogen, Carlsbad, CA, USA). Cells were incubated at 37 °C in a humidified, 5% CO_2_ incubator. All cell lines were tested for the presence of mycoplasma using polymerase chain reaction (PCR).

### 2.2. Chemicals and Antibodies

PCL (M_n_ = 700,000–900,000), sodium orthovanadate, sodium fluoride, β-glycerophosphate, DOX (doxorubicin), and BrdU were purchased from Sigma-Aldrich (St. Louis, MO, USA). Pimonidazole hydrochloride was obtained from Hypoxyprobe Inc. (Burlington, MA, USA). The following antibodies were used for the study: Alexa Fluor 594 phalloidin (Thermo Fisher Scientific, Waltham, MA, USA), anti-HIF-1α (R&D Systems, Minneapolis, MN, USA), anti-β-actin (Sigma-Aldrich, St. Louis, MO, USA), anti-F-actin (Santa Cruz Biotechnology, Dallas, TX, USA), anti-BrdU (Thermo Fisher Scientific, Waltham, MA, USA), anti-pimonidazole (Hypoxyprobe Inc.), anti-Ki67 (Cell Signaling Technology, Beverly, MA, USA), and cleaved caspase 3 (Cell Signaling Technology, Beverly, MA, USA). The following secondary antibodies were used: Alexa Fluor 488-conjugated anti-mouse (Thermo Fisher Scientific, Waltham, MA, USA), Alexa Fluor 594-conjugated anti-mouse (Thermo Fisher Scientific, Waltham, MA, USA), Alexa Fluor 594-conjugated anti-rabbit (Thermo Fisher Scientific, Waltham, MA, USA), and HRP-conjugated anti-mouse (Cell Signaling Technology, Beverly, MA, USA).

### 2.3. Electrospinning and Fabrication of pNFS

Porous pNFS was generated according to a previously reported method [11]. Briefly, the polymer for electrospinning was dissolved in 99.5% pure chloroform at a final concentration of 15% and stirred for 5 h to obtain a homogeneous solution. pNFS was fabricated by electrospinning (NanoNC, Seoul, Korea) using two-nozzle spinnerets with an average flow rate of approximately 8 μL/min, produced with a syringe pump. pNFS was collected onto a metallic mandrel rotating at 100 rpm at ambient temperature for 4 h. The nozzle tip-to-collector distance was set at 20 cm, with an electrical potential from the grounded collector plate of approximately 17.5 kV. pNFS thickness was measured using a high-precision caliper. The morphology of pNFS was assessed by scanning electron microscopy using an SEM4500 system (Sec, Suwon, Korea) (Supplementary Figure S1).

### 2.4. BrdU Proliferation Assay

Cells were seeded onto pNFS and incubated in a humidified 5% CO_2_/95% air incubator at 37 °C. After 3 days, pNFSs were stacked in four layers and incubated for 3 days. Twenty-four hours prior to separation, 10 μM BrdU was added to the multi-layered pNFS. Upon separation, each layer was washed three times with PBS and fixed with 3.7% PFA for 15 min. The cells were then washed three times with PBS, permeabilized with 0.1% (*v*/*v*) Triton X-100, and treated with 2 N HCl for 10 min. After washing three times with PBS, cells were incubated with a primary antibody against BrdU, followed by incubation with secondary antibody and counterstaining with DAPI. Images were obtained using a C1-Plus laser-scanning TE2000E confocal microscope (Nikon, Tokyo, Japan).

### 2.5. Hypoxia Detection Assay

Cells were seeded onto pNFS and incubated in a humidified 5% CO_2_/95% air incubator at 37 °C. After culturing cells on individual pNFSs for 3 days, pNFSs were stacked into four layers and incubated for an additional 3 days. Six hours prior to separation, pimonidazole (50 μM) was added. Upon separation, individual layers were washed three times with PBS, fixed with 3.7% PFA for 15 min, then washed again and permeabilized with 0.1% (*v*/*v*) Triton X-100. After washing three times with PBS, cells were incubated with primary antibody against pimonidazole followed by incubation with secondary antibody and counterstaining with DAPI. Images were obtained using a C1-Plus laser-scanning TE2000E confocal microscope (Nikon, Tokyo, Japan).

### 2.6. Analysis of Cell Penetration in pNFS

Cells were seeded onto pNFS and incubated in a humidified 5% CO_2_/95% air incubator at 37 °C. After incubating for 3 days, cells were fixed with 3.7% PFA for 15 min. Thereafter, cells were washed three times with PBS, permeabilized with 0.1% (*v*/*v*) Triton X-100, washed three times with PBS, and incubated in blocking solution (3% bovine serum albumin (BSA)) for 1 h at room temperature. The pNFSs were then incubated overnight at 4 °C with Alexa Fluor 594 phalloidin. Thereafter, the pNFSs were washed twice with PBS, and cell nuclei were stained with DAPI (Invitrogen, Carlsbad, CA, USA) for 2 min. The pNFSs were washed three times with PBS, mounted onto slides using mounting reagent (Invitrogen, Carlsbad, CA, USA), and analyzed using a C1-Plus laser-scanning TE2000E confocal microscope (Nikon, Tokyo, Japan).

### 2.7. RNA Isolation and qPCR

Total RNA was extracted from cells seeded onto pNFSs using the AccuZol reagent (Bioneer, Daejeon, Korea), and contaminating DNA was removed by treating with DNase I (New England Biolabs, Ipswich, MA, USA). cDNA was synthesized from total RNA (1 μg) using AccuPower RT PreMix (Bioneer, Daejeon, Korea) and then PCR-amplified using appropriate primers for *Glut1*, *CA9*, *VEGF*, *LDHA*, and 18S rDNA (Bioneer, Daejeon, Korea). Quantitative PCR (qPCR) was performed on a CFX Connect Real-Time PCR Detection System (Bio-Rad, Hercules, CA, USA) using iQ SYBR Green Supermix (2×) (Bio-Rad).

### 2.8. Irradiation

Cells were exposed to X-ray radiation at a dose rate of 0.72 Gy/min using an X-ray irradiator (Model X-RAD Ir160; Precision X-Ray Inc., North Branford, CT, USA).

### 2.9. Immunoblot Analysis

Cell lysates were prepared by lysis with RIPA buffer containing a protease inhibitor cocktail (Roche Applied Science, Branford, CT, USA), sodium orthovanadate, sodium fluoride, and β-glycerophosphate. Proteins in whole-cell lysates were separated by sodium dodecyl sulfate-polyacrylamide gel electrophoresis (SDS-PAGE) and transferred to nitrocellulose membranes (Bio-Rad, Hercules, CA, USA). The membranes were blocked with PBS containing 2% nonfat dry milk and incubated at room temperature with the appropriate primary and HRP-conjugated secondary antibodies. β-Actin was used as a loading control. Signals were detected using enhanced chemiluminescence reagents (Thermo Fisher Scientific, Waltham, MA, USA).

### 2.10. Analysis of Dead Cells

Cells were seeded onto pNFSs and incubated in a humidified 5% CO_2_/95% air incubator at 37 °C. After 3 days, pNFSs were stacked in four layers and incubated for 3 days. During the latter 3-day incubation period, designated samples of 4-layer pNFSs were separated into individual layers on each day, and cells in each layer were analyzed for viability using a LIVE/DEAD Fixable Green Dead Cell Stain Kit (Thermo Fisher Scientific, Waltham, MA, USA). The fluorescence of dead (green) cells was analyzed using a C1-Plus laser-scanning TE2000E confocal microscope (Nikon, Tokyo, Japan).

### 2.11. TUNEL Assay

Cells were seeded onto pNFS and incubated in a humidified 5% CO_2_/95% air incubator at 37 °C. After 3 days, pNFSs were stacked in four layers and incubated for 1 day, after which cells were treated with 3 μM DOX or exposed to ionizing radiation (4 Gy). After 1 day, cells were fixed with 3.7% PFA for 15 min, washed with PBS containing 3% (*w*/*v*) BSA, and permeabilized with 0.1% (*v*/*v*) Triton X-100. After washing with PBS, cells were incubated for 1 h at 37 °C in the dark with an apoptosis-detection solution (Apoptosis Detection System Kit; Roche Molecular Biochemicals, Mannheim, Germany). In situ-labeled nuclei were observed and imaged using a C1-Plus laser-scanning TE2000E confocal microscope (Nikon, Tokyo, Japan).

### 2.12. Immunofluorescence and Confocal Microscopy

Cells seeded onto pNFSs were fixed with 3.7% PFA for 15 min, washed three times with PBS, and permeabilized with 0.1% (*v*/*v*) Triton X-100. After 15 min, the pNFSs were washed three times with PBS and incubated in a blocking solution (PBS/3% BSA) for 1 h at room temperature. The pNFSs were then incubated overnight at 4 °C with an appropriate primary antibody. Thereafter, the pNFSs were washed three times with PBS and incubated with Alexa Fluor 594-conjugated secondary antibodies for 1 h. The pNFSs were washed twice with PBS and cell nuclei were counterstained with DAPI (Invitrogen, Carlsbad, CA, USA) for 2 min. The pNFSs were washed three times with PBS, mounted onto slides using mounting reagent (Invitrogen), and analyzed using a C1-Plus laser-scanning TE2000E confocal microscope (Nikon, Tokyo, Japan).

### 2.13. Immunohistochemistry

Ki67 and cleaved caspase 3 were detected immunohistochemically using a Vectastain Elite ABC kit (Vector Laboratories Inc., Burlingame, CA, USA) as described by the manufacturer. For immunoperoxidase labeling, endogenous peroxidase was blocked by incubating with 0.1% H_2_O_2_ in PBS for 10 min at room temperature. The samples were then incubated overnight at 4 °C with anti-Ki67 or anti cleaved caspase 3 primary antibody. After washing with PBS, samples were incubated with secondary antibody and peroxidase-antiperoxidase (PAP) complex for 30 min at room temperature. Immunoreactive sites were visualized by incubating with the HRP substrate 3,3′-DAB, and samples were counterstained with hematoxylin.

### 2.14. Statistical Analysis

All grouped data are presented as means ± SD. Differences between two groups or among multiple groups were assessed with Student’s *t*-test or analysis of variance (ANOVA), respectively, using GraphPad Prism software (GraphPad Software, San Diego, CA, USA). All experiments were repeated in at least duplicate with triplicate technical replicates.

## 3. Results

### 3.1. Characterization of Colon Cancer Cell Cultures in a Single-Layer pNFS

**Figure 1:**
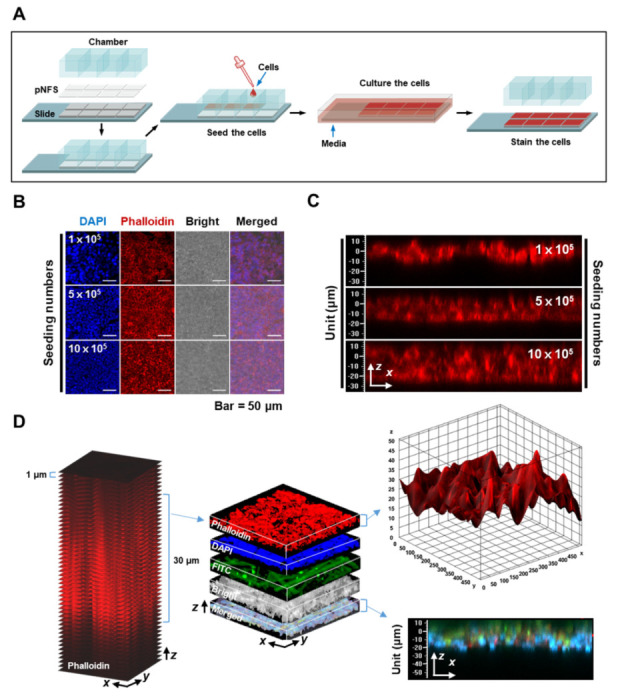
Characterization of colon cancer cells cultured in a single layer of pNFS. (**A**) Schematic workflow depicting the setup of a single-layer pNFS in an eight-well chamber slide for colon cancer cell culture and analysis. (**B**,**C**) HCT116 cells were incubated for 3 days at different densities on an eight-well chamber slide setup containing a single layer of pNFS. Confocal images show *x*- and *y*-axis (**B**) and *x*- and *z*-axis (**C**). (**D**) HCT116 cells were incubated for 3 days at different densities on an eight-well chamber slide setup containing a single layer of pNFS coated with collagen and FITC. Left panel: *z*-stack confocal images of Alexa Fluor 594 phalloidin-stained HCT116 cells; middle panel: merged *z*-stack confocal images; right upper panel: surface plot of merged z-stack confocal images of Alexa Fluor 594 phalloidin-stained HCT116 cells; right lower panel: *x*-axis and *z*-axis confocal views of the merged image.

The characteristics of colon cancer cells cultured using a single layer of pNFS are shown in Figure 1A. The single-layer culture system was established by first mounting a 30 μm-thick pNFS, wetted with 70% ethanol, onto a detachable eight-well chamber slide. The pNFS was dried on a clean bench for 16 h with UV treatment and then wetted with media at 37 °C in a humidified 5% CO_2_ incubator. After a 24-h incubation, HCT116 cells were seeded at different densities (1, 5, or 10 × 10^5^ cells/well) in a single layer of pNFS. The cells were incubated at 37 °C in a humidified 5% CO_2_ environment for 3 days, fixed with 3.7% paraformaldehyde (PFA), and then stained with Alexa Fluor 594-conjugated phalloidin and 4′,6-diamidino-2-phenylindole (DAPI). We found that, upon seeding at a density of 10 × 10^5^ cells/well, the cancer cells formed tightly packed 3D cultures within a 30 μm-thick pNFS (Figure 1B).

Next, we employed confocal z-stack microscopy, which uses optical slicing to partition the sample in the z-direction, to confirm that colon cancer cells formed tightly packed 3D cultures inside the pNFS. As shown in Figure 1C, which depicts confocal z-stack images of different 1 μm-thick planes (stacked from top to bottom) and the reconstructed 3D projection image in each pNFS, colon cancer cells seeded at a density of 10 × 10^5^ cells/well were found to fill the pNFS and grow. We then confirmed the acquired data by staining the pNFS with FITC (fluorescein isothiocyanate). Specifically, colon cancer cells (10 × 10^5^ cells/well) were seeded in a single layer of FITC-stained pNFS, incubated at 37 °C in a humidified 5% CO_2_ incubator for 3 days, and fixed with 3.7% PFA. The colon cancer cells were then immunostained with Alexa Fluor 594-conjugated phalloidin, counterstained with DAPI, and analyzed using confocal z-stack microscopy. As shown in Figure 1D, the colon cancer cells were localized to a single layer of FITC-stained pNFS. Collectively, these results suggest that the pNFS is capable of 3D culturing cells at a high density.

### 3.2. Characterization of Colon Cancer Cell Cultures in a Multi-Layer pNFS

**Figure 2:**
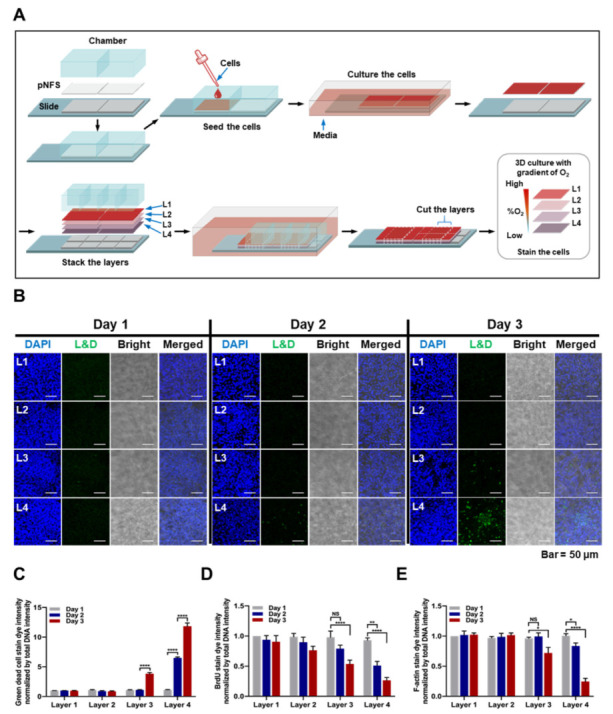
Characterization of colon cancer cells cultured in a multi-layer pNFS. (**A**) Schematic workflow showing the setup of a multi-layer pNFS in an eight-well chamber slide for colon cancer cell culture and analysis. (**B**) Live/dead cell analysis of colon cancer cells incubated in a multi-layer pNFS for 3 days. (**C**) Quantification of live/dead cells among colon cancer cells cultured in a multi-layer pNFS for 3 days. Fluorescence intensity of dead cells (green) was normalized to that of DAPI. Data are presented as means ± SD (**** *p* < 0.0001; ANOVA). (**D**) Quantification of proliferating colon cancer cells cultured in a multi-layer pNFS for 3 days. Fluorescence intensity of BrdU-stained proliferating colon cancer cells (red) was normalized to that of DAPI. Data are presented as means ± SD (** *p* < 0.01, **** *p* < 0.0001; ANOVA). NS, not significant. (**E**) Quantification of F-actin in colon cancer cells cultured in a multi-layer pNFS for 3 days. Fluorescence intensity of F-actin (red) in colon cancer cells was normalized to that of DAPI. Data are presented as means ± SD (* *p* < 0.05, **** *p* < 0.0001; ANOVA). NS, not significant.

Next, we investigated whether a colon cancer cell-culturing multi-layer pNFS (Figure 2A) could mimic the hypoxic tumor microenvironment. The first steps in establishing the multi-layer pNFS (wetting with ethanol, mounting onto a detachable two-well chamber slide, drying, and wetting with media) were the same as those for the single-layer pNFS. After incubating the setup in a humidified 5% CO_2_ incubator for 24 h, HCT116 cells were seeded in a single layer of pNFS at a single density (4 × 10^6^ cells/well) and incubated at 37 °C for 24 h. A single layer of pNFS fixed on a glass slide was separated from the chamber, immersed in media, stacked, and incubated at 37 °C in a humidified 5% CO_2_ incubator. It has been reported that a hypoxic environment forms in cancer tissue located more than 70 μm away from blood-supplying vessels [22]. Therefore, we hypothesized that multi-layer pNFS-cultured colon cancer cells situated at least 70 μm from the media would experience a hypoxic environment. Assuming that each pNFS layer is 30 μm thick, at least three layers would be required to achieve this spatial separation. In this study, we opted for a four-stack system. Specifically, colon cancer cells were cultured in four stacked layers of pNFS for 3 days, during which the stacks were separated daily for analysis of changes in cell survival and growth. To determine the survival of colon cancer cells, we performed LIVE/DEAD cell assays, measuring fluorescence of dead cells under a confocal microscope. As shown in Figure 2B, on day 1, no dead cells were observed in layer 1 (L1) to layer 4 (L4). On day 2, a few dead cells were observed in L4, farthest from the media (Figure 2B). On day 3, a few dead cells were newly observed in layer 3 (L3), and a large number of dead cells were detected in L4 (Figure 2B). Quantification of this decrease in viability, determined by measuring the intensity of dead cell fluorescence (green) relative to that of DAPI fluorescence, is shown in Figure 2C. Next, we used BrdU incorporation to investigate cancer cell proliferation. Proliferating colon cancer cells were detected in the top layer adjacent to the medium, but the number of dividing cells decreased as the distance from the medium increased (Figure 2D and Supplementary Figure S2). These results suggest that cancer cells in L3 and L4 became hypoxic on days 2 and 3. Hypoxia is known to disrupt actin dynamics and induce disorganization of the F-actin structure [23]. Thus, we investigated hypoxia-induced disorganization of F-actin by analyzing intracellular F-actin staining using a fluorescence confocal microscope. On days 1 and 2, there were no differences in the fluorescence of F-actin between layers, whereas on day 3, F-actin fluorescence decreased slightly in L3 and more significantly in L4 (Figure 2E and Supplementary Figure S3). Collectively, these results indicate that the oxygen supply and viability of colon cancer cells cultured using multilayer pNFS are increased as the pNFS layer becomes closer to the medium.

### 3.3. PNFS-Based Multi-Layer Colon Cancer Cell Culture Mimics a Hypoxic Tumor Microenvironment

**Figure 3:**
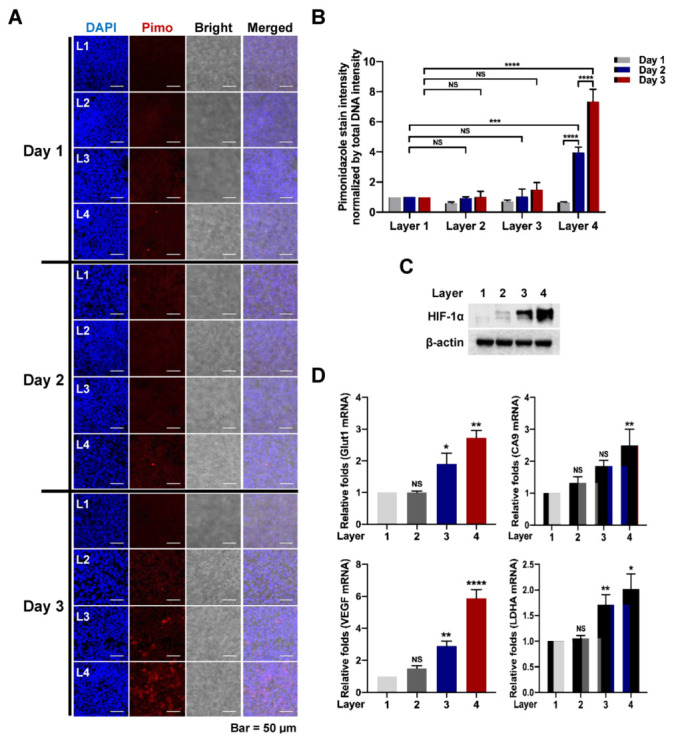
pNFS-based multi-layer colon cancer cell culture mimics the hypoxic tumor microenvironment. (**A**) Analysis of hypoxic colon cancer cells incubated in a multi-layer pNFS for 3 days. (**B**) Quantification of the fluorescence intensity of pimonidazole in colon cancer cells incubated in a multi-layer pNFS for 3 days. Fluorescence intensity of pimonidazole (red) in cancer cells was normalized to that of DAPI. Data are presented as means ± SD (*** *p* < 0.001, **** *p* < 0.0001; ANOVA). NS, not significant. (**C**) Expression of HIF-1α in colon cancer cells incubated in a multi-layer pNFS for 3 days. (**D**) Expression of HIF-1α target genes (*Glut1*, *CA9*, *VEGF*, and *LDHA*) in cancer cells incubated in a multi-layer pNFS for 3 days. Data are presented as means ± SD (* *p* < 0.05, ** *p* < 0.01, **** *p* < 0.0001; ANOVA). NS, not significant.

To confirm that the multi-layer pNFS mimics a hypoxic tumor microenvironment, we next investigated changes in oxygen supply in each layer of multi-layer pNFS cultures using pimonidazole staining; we also studied the molecular biology of colon cancer cells exposed to a hypoxic environment. A four-stack multi-layer pNFS system, seeded with HCT116 cells (4 × 10^6^ cells/well), was established as described above, and then incubated in a humidified 5% CO_2_ incubator for 3 days. On each day, the four layers of the pNFS were stained with pimonidazole (50 μM) for 6 h, and then the four layers of pNFS were separated into one layer. The immunofluorescence of pimonidazole-stained colon cancer cells was then analyzed using an anti-pimonidazole antibody. As shown in Figure 3A, on days 1 and 2, no pimonidazole-stained colon cancer cells were observed in any layer (L1 to L4). On day 3, a few pimonidazole-stained colon cancer cells were observed in L3, whereas a large number of such cells were observed in L4. Quantification of the red fluorescence intensity of pimonidazole-stained colon cancer cells relative to that of DAPI is shown in Figure 3B. We also investigated the expression of hypoxia-responsive genes in cancer cells in each layer of the multi-layer pNFS culture system. This was accomplished by separating each layer of the multiple layers of pNFS after incubation for 3 days and then extracting protein and RNA from each layer. It has been reported that members of the HIF family are essential hypoxia-inducible transcription factors that regulate adaptive cellular responses to low O_2_ concentrations in metazoans [24,25,26,27]. Therefore, we analyzed the expression of HIF-1α and its target genes, *Glut1*, *CA9*, *VEGF*, and *LDHA*, in each layer of the multi-layer pNFS. As shown in Figure 3C, HIF-1α expression increased from L1 to L4 as the distance from the medium increased, in association with an increase in the expression of HIF-1α target genes (Figure 3D and Supplementary Figure S4). Next, using HeLa cells, we investigated that the multi-layer pNFS mimics a hypoxic tumor microenvironment. Consistently, we found that HeLa cells exhibited similar data to HCT116 cells (Supplementary Figures S5 and S6). Hence, these results suggest that a multi-layer pNFS cancer cell culture system effectively mimics the hypoxic tumor microenvironment.

### 3.4. PNFS-Based Multi-Layer Colon Cancer Cell Culture for Bioassay

**Figure 4:**
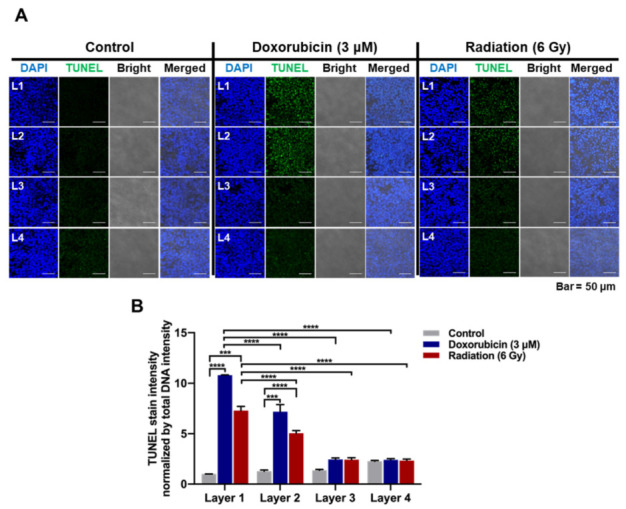
pNFS-based multi-layer colon cancer cell culture for bioassay. (**A**) Analysis of DOX or radiation-induced cell death in colon cancer cells incubated in a multi-layer pNFS for 1 day. (**B**) Quantification of apoptotic cells in DOX-treated or radiation-exposed colon cancer cells incubated in a multi-layer pNFS. Fluorescence intensity of TUNEL-positive colon cancer cells (green) was normalized to that of DAPI. Data are presented as means ± SD (*** *p* < 0.001, **** *p* < 0.0001; ANOVA).

Next, we investigated whether multi-layer colon cancer cell cultures based on pNFS can be used for bioassays. Hypoxia in solid tumors leads to resistance to various classes of chemotherapeutic agents, including anthracyclines, anthracenediones, and epipodophyllotoxins [28]. Furthermore, the sensitivity of cells or tissues to ionizing radiation decreases in hypoxia [22]. Therefore, in this study, we investigated whether mimicking hypoxia in multi-layer cultures of colon cancer cell in pNFS provides a platform for bioassaying the development of chemo- and radio-resistance in colon cancer cells. To this end, 1-day old, four-layer NFS cultures were treated with or without 3 μM doxorubicin (DOX) or ionizing radiation (4 Gy). After a 24 h incubation, the four layers of pNFS were separated into one layer, and then apoptotic cells were analyzed using TUNEL (terminal deoxynucleotidyl transferase dUTP nick-end labeling) assays. As shown in Figure 4A,B, no DOX- or ionizing radiation-induced cell death was observed in either L3 or L4. In L1, nearest the medium, a large number of apoptotic cells were observed, whereas in L2 the number of apoptotic cells was slightly reduced compared with that in L1. These results provide evidence that pNFS-based multi-layer cancer cell cultures are useful for bioassay studies, for drug screening, and as an alternative to small animals for hypoxic tumor microenvironment tests.

Immunohistochemistry (IHC), a widely used technique in many biological research fields, is a method for identifying and localizing a protein within a tissue through microscopic visualization. Here, we investigated the applicability of IHC to pNFS-based multilayer colon cancer cell cultures. In one previous report, transplanted and harvested tissue-engineered constructs were fixed in formalin and embedded in paraffin using PCL/PLLA fibrous scaffolds, after which tissues were analyzed by IHC [29]. However, another study reported that histological processing of thermosensitive PCL/PLL scaffolds fails because of the low-melting temperature (T_m_ ≈ 60 °C) characteristic of PCL [30]. To circumvent this problem, the authors combined PLC with a low-melting-point paraffin-embedding method for histological investigations of thermosensitive specimens [30]. Unfortunately, we found that embedding in low-melting-point paraffin was not suitable for preparing sections of pNFS-based multi-layer cancer cell cultures. Therefore, we developed an IHC method that utilizes thermosensitive PCL-based NFS. A four-stack multi-layer pNFS system, seeded with HCT116 cells (4 × 10^6^ cells/well), was established as described above, and then incubated in a humidified 5% CO_2_ incubator for 3 days. The four layers of pNFS were separated into one layer, fixed in ice-cold acetone for 10 min, and dried at room temperature.

Next, blocking buffer (PBS containing 10% fetal bovine serum) was added to the sample, after which cancer cell proliferation was assessed using the modified IHC method. Using the proliferation marker Ki67 or apoptotic marker cleaved caspase 3 (cc3), we detected proliferating colon cancer cells in the top layer adjacent to the medium but found that the number of proliferating cells decreased as the distance from the medium increased (Supplementary Figure S7). Collectively, our findings indicate that a pNFS-based multi-layer colon cancer cell culture system is suitable for IHC applications, and that the modified IHC method described here is useful for histological investigations of thermosensitive PCL-based specimens.

## 4. Discussion

In the present study, we demonstrated that a pNFS-based system is a suitable platform for 3D culture of colon cancer cells with a high density similar to that seen in tumor tissues and that such pNFS-based multi-layered colon cancer cell cultures mimic the hypoxic tumor environment. This simple procedure provides a versatile and experimentally convenient solution to the problem of creating 3D structures for the growth of cancer cells and tumor tissue.

As previously noted, 2D monolayer cell culture systems have been used traditionally to investigate human biology and develop therapeutics [1,2]. However, these 2D systems cannot adequately represent the structure, function, and physiology of cells in the natural 3D environment of a living tissue [3,4,5,6]. The need to solve these problems has led to the development of 3D cell culture systems [1,3,4,7].

Cell spheroids and scaffolds are the most popular in vivo tissue-mimicking 3D cell culture systems [31]. Previous reports have characterized spheroids as efficient 3D cell culture systems that mimic epithelial cancer formation and endothelial cell angiogenesis processes [31]. However, accurately reconstructing the precise 3D locations of cells in spheroids remains difficult owing to cellular heterogeneity, uncontrollable cell numbers, and necrosis caused by insufficient nutrients [4].

Bell et al., working in the 1980s, were the first tissue engineers to perform a bi-layered skin graft [19,32]. Tissue engineering techniques generally require the use of porous scaffolds to generate 3D specimens in vitro and in vivo for initial cell attachment and subsequent tissue formation [33]. FDA-approved devices and implants made from synthetic polymers, such as sutures and meshes, have been used in soft-tissue engineering [19]. New techniques have been developed based on either heating macromolecules or dissolving them in a suitable organic solvent [19]. Most currently available porous scaffolds are synthesized from polymers such as polycaprolactone and poly (lactic acid-glycolic acid) acid and are generally used for tissue-engineering research, although their use for implantation of synthetic polymer-based scaffolds of 3D cell cultures is increasing [31].

Microfabricated, porous, scaffold-based 3D cell culture systems have been proposed as an approach for addressing some of the problems of 2D cell culture systems and developing methods applicable to drug screening [1,11,19]. However, most microfabrication approaches require instrumentation that is not commonly available in biological laboratories. This has led to the development of mats consisting of fabricated and patterned biodegradable NFSs, which are readily available to biological laboratories, for cell cultures [11,20,21]. pNFS provides sufficient cell infiltration to allow a stable 3D structure and promote 3D adhesion, spreading, migration, and increased activity of dendritic cells in co-culture with cancer cells [11]. In accordance with previous reports, we found that a variety of cancer cells could infiltrate into pNFS, allowing such cells to be grown to a high density similar to that in a tumor tissue (Figure 1B,C). As shown in Figure 1D, cancer cells, identified by FITC staining, indeed filled the pNFS. To develop a 3D culture method that is better suited to biological laboratories, we used pNFS mounted onto eight-well chambers (Figure 1A) and demonstrated the possibility of generating a 3D culture of cancer cells with a tissue-like density using this approach.

A previous report suggested that the hypoxic tumor environment could be mimicked using paper-based multi-layered cancer cell cultures [1]. Seeking to extend these findings, we hypothesized that multi-layer pNFS could also be used to simulate hypoxic tumor microenvironments. We investigated that cell growth was suppressed, entry into S-phase was decreased, and DNA damage was higher in layers farther from the media compared with the top layer closest to the media. We observed that colon cancer cells cultured in multi-layer pNFS for 3 days showed an increase in cell death and a decrease in S-phase entry as the distance from the media increased (Figure 2B,C). Based on these results, we hypothesized that, by days 2 and 4, the colon cancer cells in L3 and L4 were exposed to a hypoxic environment. Consistent with a previous report that hypoxia induces disorganization of F-actin in cells [23], we observed F-actin disorganization in colon cancer cells in L3 and L4 of the multi-layer pNFS (Figure 2E and Supplementary Figure S2). Using pimonidazole immunostaining and expression of HIF-1α and its target genes as hypoxia markers, we investigated the oxygen supply per layer, confirming that multi-layer pNFS induces a hypoxic tumor microenvironment. Specifically, we found that a hypoxic tumor microenvironment is formed at distances greater than 70 μm from the oxygen supply media in the case of the multi-layer pNFS. This is supported by analyses performed on day 3, which demonstrated the presence of a few pimonidazole-stained cancer cells in L3 and a large number of such cells in L4 (Figure 3A,B and Supplementary Figure S5A,B), as well as an increase in the expression of HIF-1α and its target genes, *Glut1*, *CA9*, *VEGF*, and *LDHA*, as the distance from the media increased (i.e., from L1 to L4). In this study, HCT116 cells and HeLa cells were used. As cell lines have different shapes, sizes, and growth rates, we will perform research using various cell lines in the future. These results suggest that a multi-layered pNFS colon cancer cell culture system can mimic the hypoxic tumor microenvironment. We further found that multi-layer colon cancer cell cultures based on pNFS could be using for bioassays, showing that DOX or ionizing radiation induced extensive apoptosis in the nearest layer (L1) and somewhat lesser cell death in the adjacent layer (L2), but caused no cell death in the most distant layers, L3 and L4 (Figure 4A,B). Collectively, these results indicate that a multi-layer pNFS with in situ-cultured colon cancer cells effectively mimics the hypoxic tumor microenvironment and further reveal an in vivo-like increase in the chemo- and radio-resistance of colon cancer cells in the hypoxic layers of pNFS.

IHC, a basic technique used in many fields of biological research, is a method for identifying localization of a protein within tissue through microscopic visualization. As the low melting temperature (60 °C) of the PCL scaffold makes it difficult to insert PCL-based NFS into paraffin, a PCL-based NFS is not suitable for IHC [30]. In this study, we circumvented this limitation, developing a modified IHC method for histological investigation of heat-sensitive PCL-based specimens that is suitable for pNFS-based multilayered colon cancer cell cultures (Supplementary Figure S7).

In this study, the movement of fluid stopped in the 3D culture. However, fluid continuously moves in the actual cancer environment. In the future, to mimic the exact hypoxic tumor microenvironment, we plan to apply microcirculation to the medium and measure the degree of hypoxia in each layer in the scaffold.

In conclusion, we suggest that pNFS-based multi-layer cell cultures can serve as a useful, versatile, and convenient tool for basic cell biology investigations as well as tissue engineering and drug development applications.

## 5. Conclusions

Multi-layer, nanofibrous poly(ε-caprolactone) (PCL) scaffold (pNFS)-based colon cancer cell cultures mimic the hypoxic tumor microenvironment. This pNFS-based multi-layered colon cancer cell culture system is useful for basic cell biology investigations as well as tissue engineering and drug development applications.

## Data Availability

Data presented in this study are available in the article or Supplementary Materials or upon request to the corresponding author.

## Supplementary Materials

The following are available online at https://www.mdpi.com/article/10.3390/cancers13143550/s1, Figure S1: SEM images of the single layer of pNFS. Figure S2: Analysis of BrdU incorporation of cells among the cancer cells incubated in the multi-layer of pNFS for three days. Figure S3: Analysis of F-actin of cells among the cancer cells incubated in the multi-layer of pNFS for three days. Figure S4: Uncropped images related to Figure 3C. Figure S5: pNFS-based multi-layer cancer cell (HeLa cell) culture mimics the hypoxic tumor microenvironment. Figure S6: Uncropped images related to Supplementary Figure S5C. Figure S7: Immunohistochemical analysis of pNFS-based multi-layer cancer cell culture.
