# Supplementary material for: Multi-Layer Nanofibrous PCL Scaffold-Based Colon Cancer Cell Cultures to Mimic Hypoxic Tumor Microenvironment for Bioassay
Source: Cancers (Basel). 2021 Jul 15;13(14):3550. doi: 10.3390/cancers13143550 (PMC8305385; doi:10.3390/cancers13143550)
Supplement: Supplementary file 1 [file cancers-13-03550-s001.zip › cancers-1188584-supplementary.pdf]

Multi-layer nanofibrous PCL scaffold-based cancer cell cultures to mimic hypoxic tumor microenvironment for bioassay

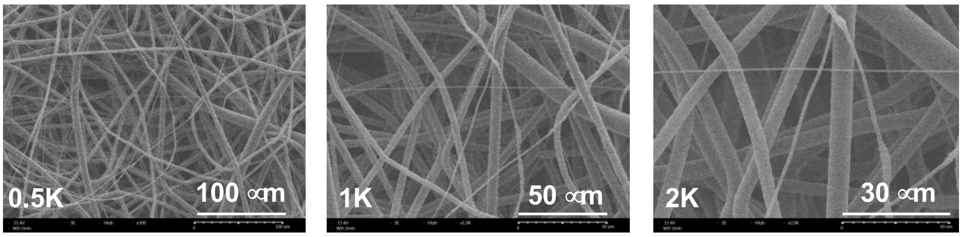

Supplementary Figure S1. SEM images of the single layer of pNFS.

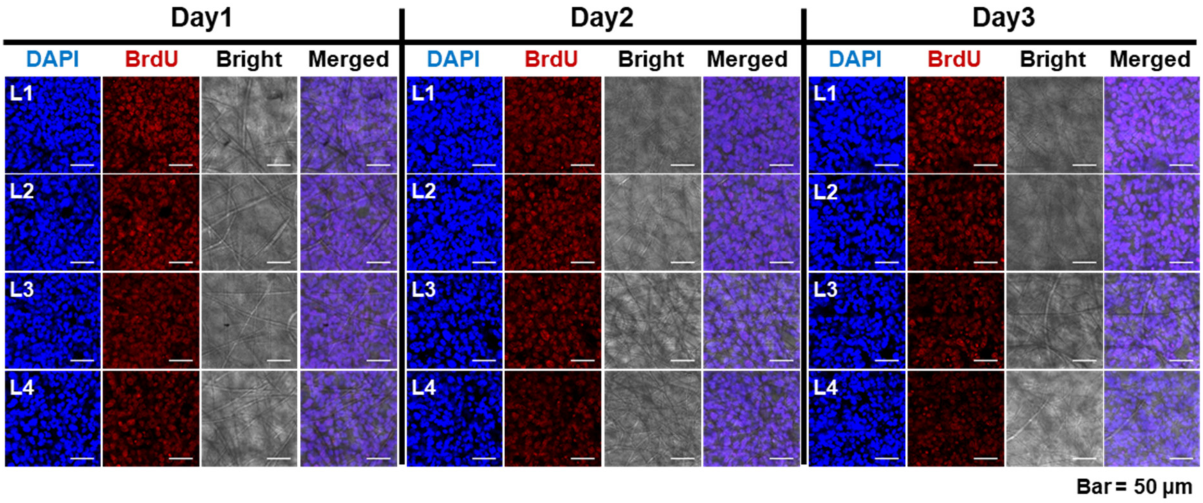

Supplementary Figure S2. Analysis of BrdU incorporation of cells among the cancer cells incubated in the multi-layer of pNFS for three days.

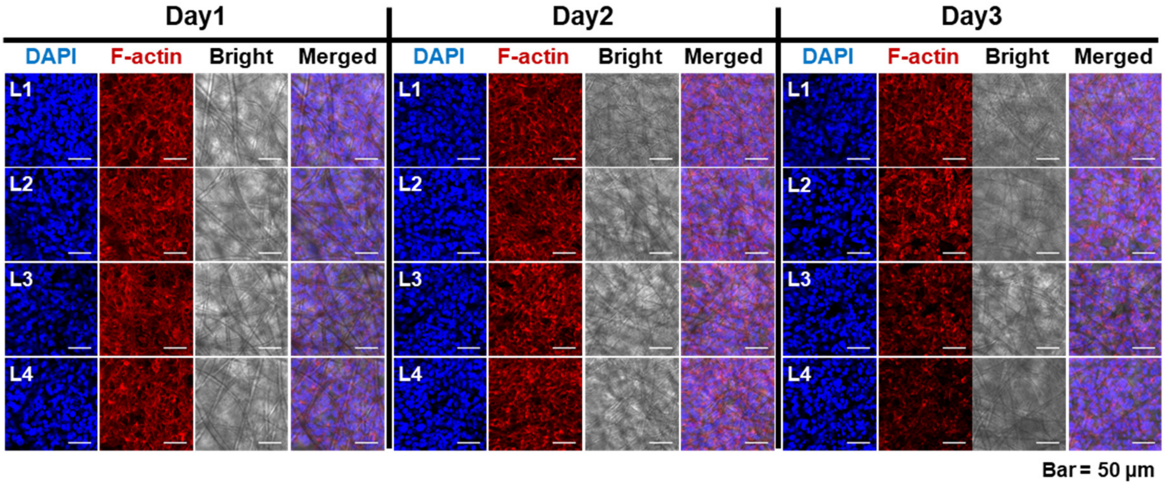

Supplementary Figure S3. Analysis of F-actin of cells among the cancer cells incubated in the multi-layer of pNFS for three days.

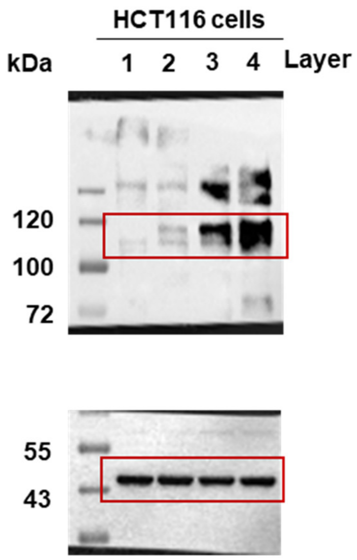

Supplementary Figure S4. Uncropped images related to Figure. 3C.

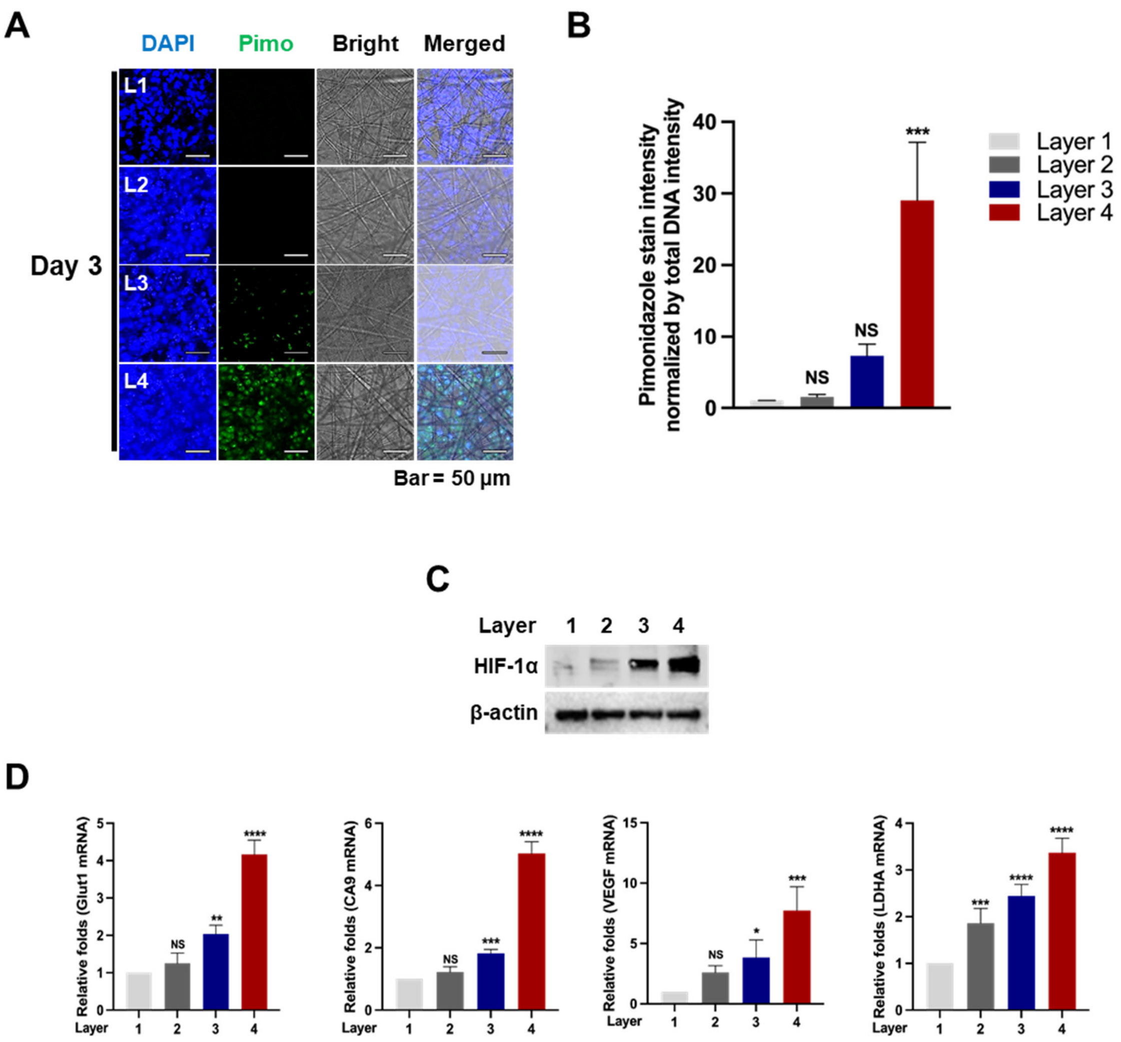

Supplementary Figure S5. pNFS-based multi-layer cancer cell (HeLa cell) culture mimics the hypoxic tumor microenvironment. (A) Analysis of hypoxic cancer cells

incubated in a multi-layer pNFS for 3 days. (B) Quantification of fluorescence intensity of pimonidazole in cancer cells incubated in a multi-layer pNFS for 3 days. Fluorescence intensity of pimonidazole (green) in cancer cells was normalized to that of DAPI. Data are presented as means ± SD (***$p < 0.001$; ANOVA). NS, not significant. (C) Expression of HIF-1α in cancer cells incubated in a multi-layer pNFS for 3 days. (D) Expression of HIF-1α target genes (*Glut1*, *CA9*, *VEGF*, and *LDHA*) in cancer cells incubated in a multi-layer pNFS for 3 days.

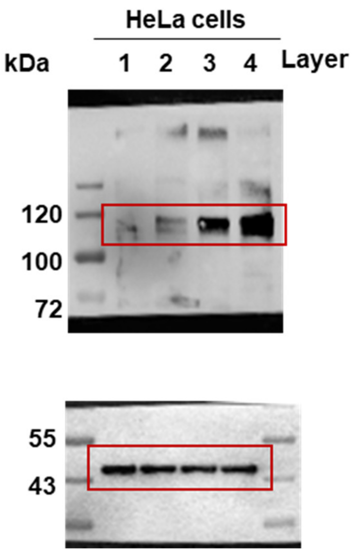

Supplementary Figure S6. Uncropped images related to Supplementary Figure. 5C.

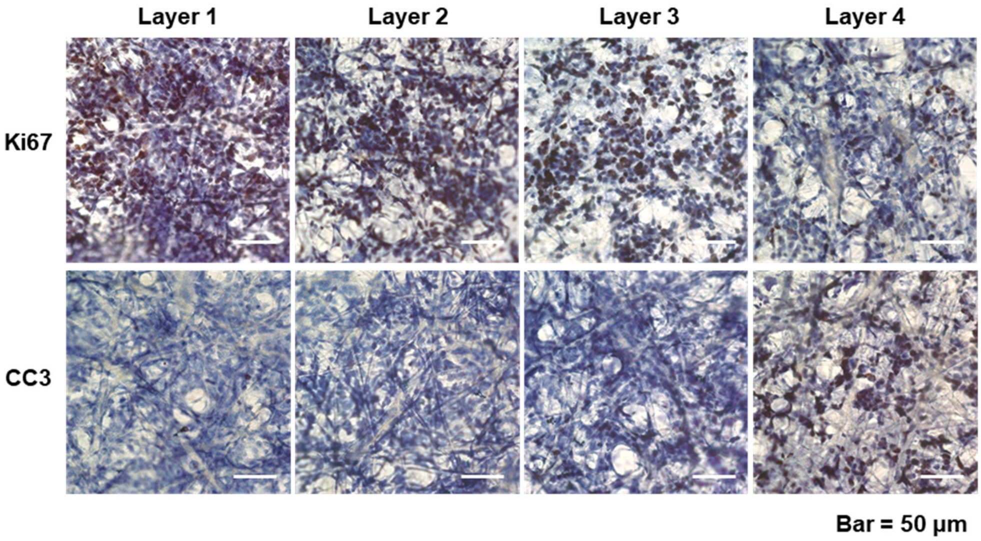

Supplementary Figure S7. Immunohistochemical analysis of pNFS-based multi-layer cancer cell culture. A multi-layer pNFS was stained for proliferation (Ki67) and apoptosis (CC3) markers using the HRP substrate, 3,3′-DAB. Scale bar = 50 μm.
